# Ancestrally Duplicated Conserved Noncoding Element Suggests Dual Regulatory Roles of HOTAIR in *cis* and *trans*

**DOI:** 10.1016/j.isci.2020.101008

**Published:** 2020-03-25

**Authors:** Chirag Nepal, Andrzej Taranta, Yavor Hadzhiev, Sachin Pundhir, Piotr Mydel, Boris Lenhard, Ferenc Müller, Jesper B. Andersen

**Affiliations:** 1 Biotech Research and Innovation Centre, Department of Health and Medical Sciences, University of Copenhagen, Ole Maaløes Vej 5, 2200 Copenhagen N, Denmark; 2 Institute of Cancer and Genomics Sciences, College of Medical and Dental Sciences, University of Birmingham, Edgbaston, B15 2TT Birmingham, UK; 3 Broegelmann Research Laboratory, Department of Clinical Science, University of Bergen, Bergen, Norway; 4 Department of Microbiology, Faculty of Biochemistry, Biophysics and Biotechnology, and Malopolska Centre of Biotechnology, Jagiellonian University, Karkow, Poland; 5 Institute of Clinical Sciences MRC Clinical Sciences Centre, Faculty of Medicine, Imperial College London, Hammersmith Hospital Campus, Du Cane Road, London W12 0NN, UK; 6 Sars International Centre for Marine Molecular Biology, University of Bergen, 5008, Bergen, Norway

**Keywords:** Biological Sciences, Molecular Biology, Molecular Mechanism of Gene Regulation

## Abstract

*HOTAIR* was proposed to regulate either HoxD cluster genes in *trans* or HoxC cluster genes in *cis*, a mechanism that remains unclear. We have identified a 32-nucleotide conserved noncoding element (CNE) as *HOTAIR* ancient sequence that likely originated at the root of vertebrate. The second round of whole-genome duplication resulted in one copy of the CNE within *HOTAIR* and another copy embedded in noncoding transcript of *HOXD11*. Paralogous CNEs underwent compensatory mutations, exhibit sequence complementarity with respect to transcripts directionality, and have high affinity *in vitro*. The *HOTAIR* CNE resembled a poised enhancer in stem cells and an active enhancer in HOTAIR-expressing cells. *HOTAIR* expression is positively correlated with *HOXC11 in cis* and negatively correlated with *HOXD11 in trans*. We propose a dual modality of *HOTAIR* regulation where transcription of *HOTAIR* and its embedded enhancer regulates *HOXC11 in cis* and sequence complementarity between paralogous CNEs suggests *HOXD11* regulation *in trans*.

## Highlights

• Two conserved noncoding elements (CNEs) overlap HOTAIR and its paralog on HOXD cluster
• Paralog on HOXD cluster may resolve controversy over *cis* and *trans* effects of HOTAIR
• CNEs are positively coregulated with *cis* HOX genes but negatively with *trans* cluster
• Transcribed CNEs with coevolved complementarity suggest hybridization-based function

## Graphical Abstract

**Figure:**
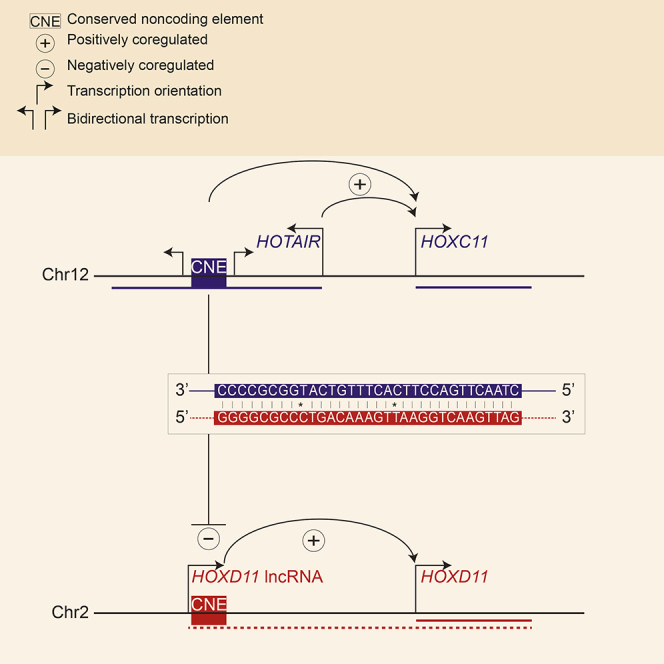

## Introduction

Mammalian genomes are pervasively transcribed, giving rise to thousands of long noncoding RNAs (lncRNAs) (Hon et al., 2017, Iyer et al., 2015). Only a handful of lncRNAs have well-characterized functions, which are attained through diverse mechanisms (chromatin regulation, alternative splicing, gene silencing, *trans*-regulation) (Guttman and Rinn, 2012, Mercer and Mattick, 2013). Although most early studies showed lncRNAs repress gene expression, some lncRNAs have enhancer-like functions and regulate genes in *cis* (Orom et al., 2010). Genomic deletion of lncRNA also removes *cis*-regulatory DNA elements, thus confounding whether the observed phenotype is due to the underlying genomic DNA, the lncRNA transcript itself, or transcription (Bassett et al., 2014, Engreitz et al., 2016, Kaikkonen and Adelman, 2018). As such, transcription blockage and perturbation of the *Lockd* lncRNA showed that it regulates *Cdkn1b* transcription through an embedded enhancer sequence, whereas the lncRNA transcript is dispensable for *Cdkn1b* expression (Paralkar et al., 2016). Deletion of 12 genomic loci encoding various lncRNAs revealed 5 loci whose deletion affected the general process of transcription and enhancer-like activity, but no requirement for the lncRNA transcripts (Engreitz et al., 2016). *Lincp21* locus previously thought to function through its RNA transcript was shown to include multiple enhancers and regulate genes in *cis* (Groff et al., 2016). Moreover, genomic and epigenomic functional annotation have revealed that most intergenic lncRNAs originate from enhancers (Hon et al., 2017). In line with enhancer function overlapping with lncRNAs, the *Haunt* lncRNA has dual roles (Yin et al., 2015), where its DNA encodes enhancers to activate HoxA genes and *Haunt* lncRNA prevents aberrant HoxA expression.

*HOTAIR* is an intergenic lncRNA located between *HOXC11* and *HOXC12* in chromosome 12. It was proposed to regulate HOXD cluster genes (i.e., *HOXD8*, *HOXD9*, *HOXD10,* and *HOXD11*; located in chromosome 2) in *trans* by recruiting the Polycomb Repressive Complex 2 (PRC2) (Rinn et al., 2007). However, this regulatory model was questioned, as PRC2 binding is promiscuous (Davidovich et al., 2013) and PRC2 was found to be dispensable for *HOTAIR*-mediated transcriptional repression (Portoso et al., 2017). Deletion of the entire Hoxc cluster (including *Hotair*) in mouse showed limited impact on gene expression and H3K27me3 levels at Hoxd genes (Schorderet and Duboule, 2011). Specific deletion of *Hotair* produced a phenotype of homeotic transformation and skeletal malformation as well as genome-wide decrease in H3K27me3 levels and upregulation of posterior HoxD genes (i.e., *Hoxd10*, *Hoxd11,* and *Hoxd13*) (Li et al., 2013). These observations were challenged as specific knockouts of the *Hotair* locus *in vivo* have shown neither homeotic transformation nor upregulation of HoxD genes, but instead a significant change in HoxC (especially *Hoxc11* and *Hoxc12*) cluster genes (Amandio et al., 2016). This strongly argues in favor of a DNA-dependent effect of the *Hotair* deletion (Amandio et al., 2016). Whether *cis*-regulation of *HOTAIR* is mediated via an unannotated enhancer element within its gene body or through transcription of the *HOTAIR* promoter remains unknown. Different regulatory mechanisms (*cis* versus *trans*) might be explained by tissue origin and changes in developmental stages in distinct genetic backgrounds (Li et al., 2016a). As such, there is no consensus model for *HOTAIR*-mediated regulation (Selleri et al., 2016). As the two current models suggest fundamentally different modes of *HOTAIR* function, we decided to revisit the role of *HOTAIR* by a systematic comparative genomic analysis.

To address whether *HOTAIR* regulates HOXC cluster genes in *cis* (Amandio et al., 2016) or HOXD cluster genes in *trans* (Li et al., 2013, Rinn et al., 2007), we exploited comparative sequence analysis across vertebrates and integrated this with transcriptomic and epigenomic data in human and mouse. The HOXC and HOXD clusters originated from an ancestral HOXC/D cluster during the second round of whole-genome duplication (WGD). We hypothesized that the two clusters may contain previously undetected remnants of an ancestral sequence, which might provide important clues on *cis* and/or *trans* interactions. We have identified and characterized a 32-nucleotide conserved noncoding element (CNE) as the *HOTAIR* ancestral sequence, which is shared by both paralogous loci in HoxC and HoxD clusters, presenting itself in an inverted syntenic position. Strikingly, the paralogous CNEs underwent compensatory mutations during vertebrate evolution, which exhibit sequence complementarity dependent on the transcript orientation. Also, the CNEs have high interaction propensity revealed by microscale thermophoresis (MST). These observations suggest direct hybridization between the two noncoding transcripts. *HOTAIR* CNE represents either an active or poised enhancer in different cellular contexts. Its expression is positively correlated with *HOXC11,* whereas negatively correlated with *HOXD11*, suggesting dual modality of *HOTAIR* CNE in *cis* and *trans*.

## Results

### Identification of *HOTAIR* Ancient Sequence and Its Paralog in HoxD Cluster

**Figure 1:**
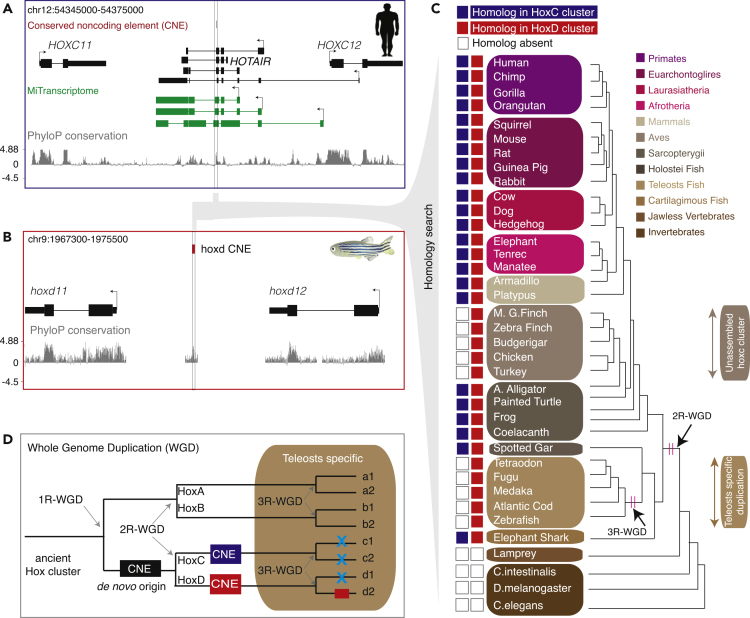
Identification of the *HOTAIR* Conserved Noncoding Element (CNE) and Its Homolog in HOXD Cluster across Vertebrates (A) A genome browser view around *HOTAIR* locus showing CNE from ANCORA browser and UCSC PhyloP conservation track. The CNE highlighted in a rectangular box is located eight nucleotides away from the splice site. (B) The ortholog of the CNE mapped to the zebrafish hoxd (between *hoxd11* and *hoxd12*) cluster. (C) Homology search of the CNE across 37 species identified homologous CNEs in only HoxC and HoxD clusters. Homologs of the CNE are undetected in jawless vertebrate and invertebrates. Homologs in HoxC and HoxD clusters are represented by blue and red, respectively. Empty boxes indicate absence of homologs. (D) Schematic representation for the proposed model of the origin of the CNE. The CNE might have a *de novo* origin in ancestral HoxC/D cluster where the second round of whole-genome duplication (2R-WGD) resulted two copies in HoxC and HoxD clusters. Teleost-specific duplication might have resulted in loss of CNE from both HoxC clusters and one of the HoxD cluster.

The Hox gene clusters are highly conserved across all vertebrates and contain multiple regulatory elements that often have small stretches of highly conserved noncoding elements (CNEs) (Engstrom et al., 2008, Lee et al., 2006). As *HOTAIR* is located within the highly conserved HoxC cluster, we asked whether it has small stretches of conserved sequences that were previously overlooked (He et al., 2011). To this end, we analyzed human and zebrafish annotated CNEs from the synteny analysis tool ANCORA (Engstrom et al., 2008) and identified a 32-nucleotide long CNE (Figure 1A) that is conserved across vertebrates (Figures S1A and S1B). Depending upon the transcript models, the CNE sequence is either located in the intron of Ensembl transcripts or in the exon of an intron-retained alternative transcript annotated in the lncRNA catalog (Figure 1A) (Iyer et al., 2015).

The CNE in zebrafish mapped between *hoxd11a* and *hoxd12a* in the hoxd cluster (Figure 1B), but not in the *hoxC* cluster. To determine whether the CNE is located in the HoxC cluster (the capitalized “HoxC” is used to represent the HoxC cluster across multiple species) or HoxD cluster (Figures S1A and S1B), we systematically mapped CNE sequences across 34 vertebrates and 3 invertebrates (Table S1). Two copies of the CNE were identified in all jawed vertebrates (except in teleosts and birds), but not in the jawless vertebrate lamprey and invertebrates (Figure 1C). The homologous CNEs mapped between *HoxD11* and *HoxD12* (reported target genes of *HOTAIR* in *trans*) in the HoxD cluster and between *HoxC11* and *HoxC12* (reported target genes of *HOTAIR* in *cis*) in the HoxC cluster (Figure 1C) in synteny, suggesting paralogy. The absence of CNE in HoxC cluster in birds might be due to the unassembled HoxC cluster (Table S2). In contrast, teleosts have well-annotated *hoxc11* and *hoxc12* genes in the same cluster (Table S2), but underwent an additional round of teleost-specific WGD, resulting in lineage-specific loss of the paralog. The basal group of jawed vertebrates, such as elephant shark (cartilaginous fish) and spotted gar (basal ray-finned fish; sister group of teleosts) (Figure 1C), have two copies of the CNE suggesting that it was already present in the ancestral HoxC/D cluster and resulted in two copies following the second round of WGD (Figure 1D). Although CNE and its flanking sequences are duplicated from the common ancestral sequence (Figure S1C), the flanking regions have limited homology (for example, in human and elephant shark; Figure S1D). However, CNE and its flanking sequences aligned separately across HoxC and HoxD clusters and revealed a relatively long stretch of sequence conserved across vertebrates (except teleosts) (Figures S1E and S1F). Thus we conclude that *HOTAIR* CNE is the ancient sequence and has two paralogous copies in all jawed vertebrates, except teleosts.

### Paralogous CNEs Are Transcribed and Embedded in Mature Noncoding Transcripts

**Figure 2:**
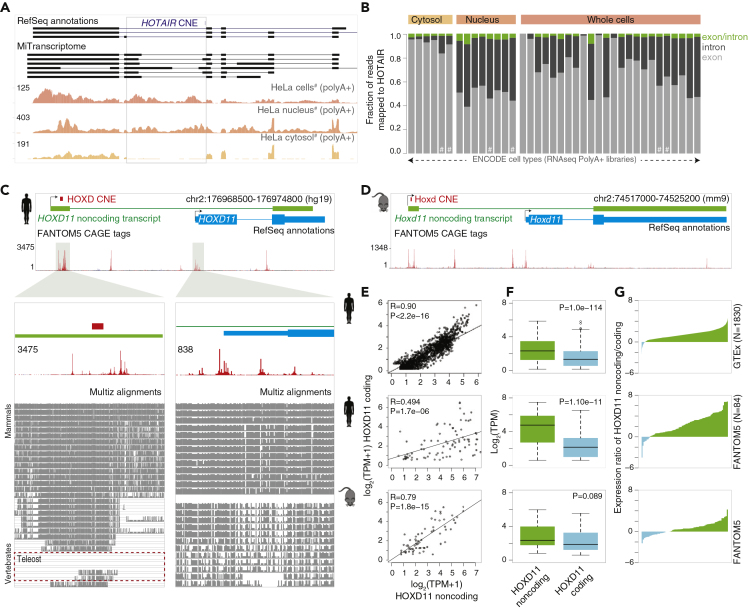
Paralogous CNEs Are Embedded in Mature Noncoding Transcripts (A) A genome browser view shows HOTAIR transcripts model from RefSeq and MITranscriptome along with RNA-seq coverage tracks from HeLa S3 cells. Large number of reads map to introns in whole cells and nuclear fraction-enriched libraries and are depleted in cytosol fraction-enriched library. (B) Distribution of reads mapped to *HOTAIR* exon, intron, and overlapping exon/intron junctions across multiple cell types. “#” denotes the HeLa S3 cells. Cell types are ordered based on increasing number of mapped reads. (C and D) A genome browser view to show transcription and sequence conservation around the *HOXD11* coding (cyan) and *HOXD11* noncoding (*ncHOXD11*) transcripts (green) in human (C) and mouse (D). The zoomed-in promoter regions show lack of sequence conservation of *ncHOXD11*. (E) Correlation of expression levels of *ncHOXD11* with *HOXD11* coding gene across multiple samples in human (from GTEx and FANTOM5 cohorts) and mouse (FANTOM5 cohort). (F) Expression levels of *ncHOXD11* and *HOXD11* coding gene across multiple samples from GTEx and FANTOM. (G) Ratio of expression levels of *ncHOXD11* and coding gene across individual cell types. Positive value on y axis indicates higher expression levels of *ncHOXD11*.

Our findings of paralogous CNE in the HoxD cluster suggest the existence of a *HOTAIR* homolog transcript overlying the CNE. To understand whether CNEs are embedded in the mature transcript, we first confirmed that *HOTAIR* CNE can be embedded in the exon by an intron-retained transcript model (Figure 1A). We analyzed long RNA sequencing (RNA-seq) data from ENCODE cell types (Djebali et al., 2012) and observed a large number of reads mapping to introns, particularly the region overlapping CNE, as shown for HeLa S3 cells (Figure 2A). A significant proportion of reads mapped to introns both in whole cell and in nuclear fraction-enriched RNA libraries and was depleted in cytosol-enriched RNA libraries (Figure 2A). We quantified reads mapped to exon, intron, and exon/intron junctions across different cell types and observed a large fraction of reads (relative to exons) mapped to introns in *HOTAIR* (Figure 2B and Table S3), but not in *HOXC11* and *HOXD11* genes (Figure S2A). We observed that a similar pattern of reads mapped to the intron in the region overlapping the CNE across different cell types (Yue et al., 2014), additionally confirming that intron retention of *Hotair* is conserved in mouse (Figures S2B and S2C). Collectively, this suggests that the *HOTAIR* CNE is embedded in an intron-retained transcript.

To associate HOXD CNE with the transcript models, we intersected it with Ensembl transcripts and identified that the CNE is embedded in the exon of a previously annotated noncoding transcript sharing the locus with *HOXD11* coding gene in human (Figure 2C) and mouse (Figure 2D). The CNE is located in the promoter region of *HOXD11* noncoding transcript (referred as *ncHOXD11* from here on), which is approximately 55 nucleotides downstream of the dominant transcription start site (denoted by the highest CAGE peak in FANTOM5 data) in human and mouse (Figures 2C and 2D). Given the shared locus, we sought to understand the nature and extent of *ncHOXD11* usage in relation to the *HOXD11* coding gene. The expression level of *ncHOXD11* is positively correlated with *HOXD11* coding gene across GTEx (GTEx Consortium, 2013) and FANTOM5 (Arner et al., 2015, FANTOM Consortium and the RIKEN PMI and CLST (DGT) et al., 2014) data in human and mouse (Figure 2E). The expression of *ncHOXD11* is significantly higher than that of the *HOXD11* coding gene (Figure 2F) in the majority of cell types in human (Figure 2G). However, in mouse, the expression of *ncHoxd11* is only marginally higher than that of the *Hoxd11* coding gene (Figures 2F and 2G), suggesting a relative gain in the expression of *ncHOXD11* in human. Finally, we analyzed RNA-seq transcript models across species (Basu et al., 2016, Hezroni et al., 2015) and detected both transcripts in ferret and dog, whereas only *ncHoxD11* in chicken (Figure S2D). The location of HoxD CNE, which is downstream of *ncHoxD11* start site, is conserved across species, suggesting that its transcription from *ncHoxD11* is an ancient phenomenon. However, *ncHoxD11* was undetected in teleosts (zebrafish and tetraodon; Figure S2D), which is further supported by absence of *ncHoxD11* promoter sequence (Figure 2A). Collectively, we showed that paralogous CNEs are transcribed and embedded in mature transcripts across multiple species.

### Transcribed CNEs Exhibit Conserved Sequence Complementarity across Vertebrates

**Figure 3:**
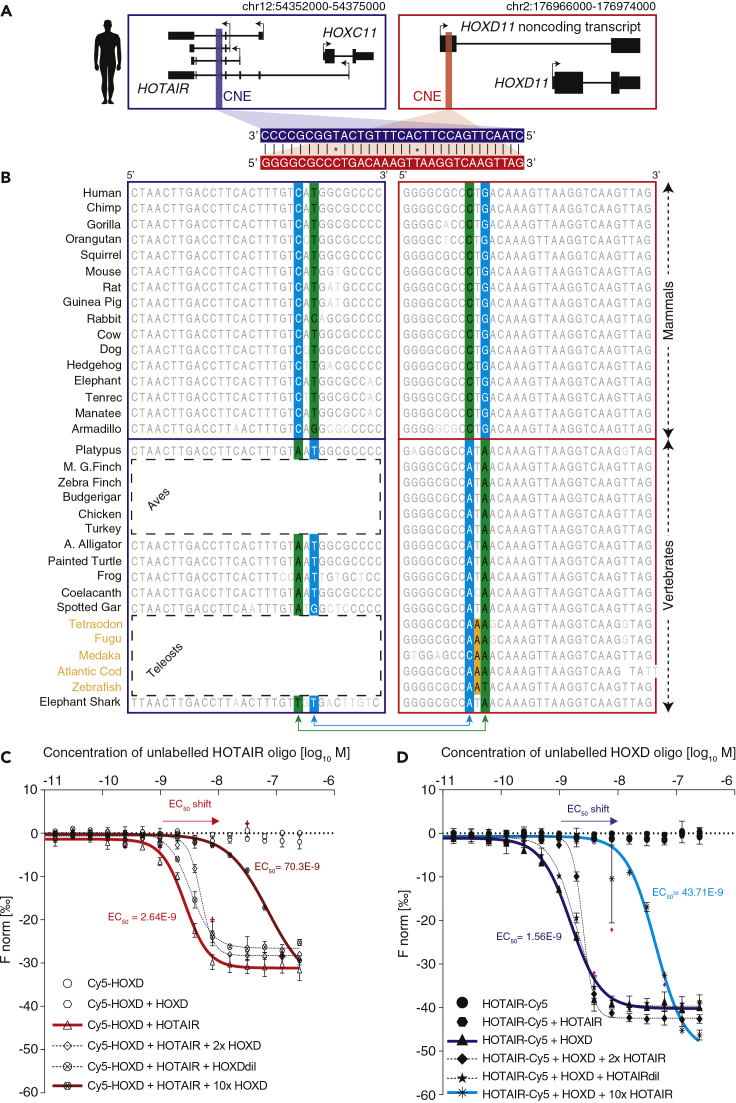
Paralogous CNEs Exhibit Sequence Complementarity in Transcript Orientation (A) Paralogous *HOTAIR* CNE (blue bar) and HOXD CNE (red bar) are zoomed and aligned in 5′ to 3′ orientation of respective transcripts. (B) Alignment of the paralogous CNEs in 5′ to 3′ orientation of respective transcripts reveals sequence complementarity across vertebrates. Genetic substitutions within paralogous CNEs co-occurred at specific positions, which resulted in gain or loss of complementarity, where green represents non-complementary DNA and cyan represents complementary DNA. Teleost-specific change in DNA sequence is shown in orange. (C and D) Microscale thermophoresis (MST) assay to evaluate the interaction between labeled and unlabeled RNA-oligos at different concentration. MST-on time of 5 s was used for analysis. Baseline-corrected normalized fluoresce (ΔF_Norm_) was chosen to present data (independent n ≥ 3 measurements; each point on the graphs presents mean ± SD). An extrapolated EC_50_ ± SD curve is fitted and shown on the graph. The concentration of the labeled RNA-oligo was constant at 5 nM. The concentration of unlabeled RNA-oligo was varied at 250 nM to 7.63 pM. The x axis represents the concentration of titrated unlabeled RNA-oligo. The y axis represents interaction-driven normalized fluorescence change (ΔFnorm[‰]). Measurement of interaction between (C) labeled HOXD CNE (Cy5-HOXD) RNA-oligo and unlabeled HOTAIR CNE RNA-oligo and (D) labeled HOTAIR CNE (HOTAIR-Cy5) RNA-oligo and unlabeled HOXD CNE RNA-oligo.

As paralogous CNEs are embedded in mature transcripts, we sought to analyze their directionality with respect to transcript orientation. We observed that human CNEs exhibit sequence complementarity in transcript orientation (Figure 3A). To ensure that the observed sequence complementarity in human is not by chance we analyzed its orientation in other vertebrates. As transcriptional evidence of *HOTAIR* and *ncHOXD11* was limited to a subset of species (Figure S2E), we inferred orientation for missing transcripts (see Methods), as illustrated for chimp and painted turtle (Figures S3A and S3B). We then aligned CNEs in the transcript orientation and observed sequence complementarity across vertebrates (Figures 3B and S3C). This suggests that sequence complementarity between CNEs is an ancient feature that has been under selection pressure for more than 300 million years. It raises an important question as to whether the key function of these transcripts is to provide transcription of the CNE.

Interestingly, in addition to the conservation and retention of sequence complementarity, we observed that paralogous CNEs revealed a specific pattern of genetic substitution at two specific positions in both CNEs that co-evolved in two separate waves in vertebrates and mammals (Figure 3B). The sequence pairs that co-evolved at two specific positions are depicted in green (non-complementary) and cyan (complementary) (Figure 3B). The nucleotides “A” colored by green in vertebrates are non-complementary, where both nucleotides co-evolved simultaneously in mammalian lineage resulting in gain of complementarity (highlighted by cyan). On the other hand, nucleotides “A” and “T” highlighted by cyan in vertebrates are complementary, where the nucleotide “A” evolved in mammals resulting in loss of complementarity. In mammals, one substitution resulted in retention of complementarity and the other substitution resulted in loss of complementarity, reflecting that paralogous CNEs underwent compensatory mutations. Unlike vertebrates, the HoxD CNE in teleosts evolved separately in its own lineage (highlighted in orange) reflecting no selection pressure to retain sequence complementarity as its putative binding partner in HoxC cluster is lost. Collectively, the coevolution of CNEs and retention of sequence complementarity in the transcript orientation raises the potential for such hybridization based on *trans* function.

### Hybridization of Paralogous CNEs *In Vitro*

To verify whether paralogous CNE transcripts hybridize, we designed two Cy5-labeled RNA-oligos (Table S4) for HOXD CNE (Cy5-HOXD) and HOTAIR CNE (HOTAIR-Cy5) and analyzed the interaction propensity using MST (Asmari et al., 2018, Duhr and Braun, 2006a, Duhr and Braun, 2006b, Moon et al., 2018). For labeled Cy5-HOXD RNA-oligo (5 nM), we analyzed the binding with unlabeled *HOTAIR* CNE RNA-oligo titrated at concentrations ranging between 250 nM and 7.63 pM. Similarly, for labeled HOTAIR-Cy5 RNA-oligo, we analyzed the binding with titrated unlabeled HOXD CNE RNA-oligo (Table S5 and Figures S4A–S4C). The labeled Cy5-HOXD and unlabeled *HOTAIR* CNE RNA-oligo showed a strong interaction at the nanomolar scale (EC_50_ = 2.64 × 10^−9^) (Figure 2C; red line), whereas we observed no binding at control conditions (either labeled oligo alone or mix of labeled and unlabeled counterparts). Similarly, the labeled HOTAIR-Cy5 and unlabeled HOXD CNE RNA-oligo showed a strong interaction at the nanomolar scale (EC_50_ = 1.56 × 10^−9^) (Figure 2D; blue line), whereas the control showed no binding. To evaluate if an unlabeled oligo can affect the interaction between labeled Cy5-HOXD RNA-oligo and the unlabeled HOTAIR CNE RNA-oligo, we added unlabeled HOXD RNA-oligo and observed that the interaction (EC_50_ = 70.3 × 10^−9^) was sensitive to the presence of unlabeled RNA-oligo (Figure 3C; dark red line). A 10-fold excess of the competitor resulted in a shift of the fluorescent signal resembling depletion of the titrated oligos and correspondingly a shift in EC_50_ value (Figure 3C; dark red line). Similarly, addition of an unlabeled *HOTAIR* CNE RNA-oligo affected the interaction of the labeled HOTAIR-Cy5 RNA-oligo and unlabeled HOXD CNE RNA-oligo, resulting in a shift in fluorescent signal (Figure 3D; cyan line). Even at low concentration of the competitor oligo the shift is still clear, confirming that the paralogous CNEs have strong interaction *in vitro*.

### Chromatin Structure of *HOTAIR* CNE Represents a Poised Enhancer in Stem Cells

CNEs are putative *cis*-regulatory elements (Bejerano et al., 2004, Harmston et al., 2013, Sandelin et al., 2004), and many of them have been experimentally validated as tissue-specific enhancers (Nobrega et al., 2003, Pennacchio et al., 2006, Woolfe et al., 2005). We analyzed experimentally validated enhancers (Pennacchio et al., 2006) and found that the genomic regions overlapping CNEs were not probed for enhancer activity. However, in the literature, we found that the region overlapping the Hoxd CNE was tested for enhancer activity in mouse and shown to drive expression in a proximal posterior part of the developing forelimbs (Beckers et al., 1996). However, subsequent deletion of Hoxd CNE revealed no phenotype *in vivo* (Beckers and Duboule, 1998) (see Discussion).

**Figure 4:**
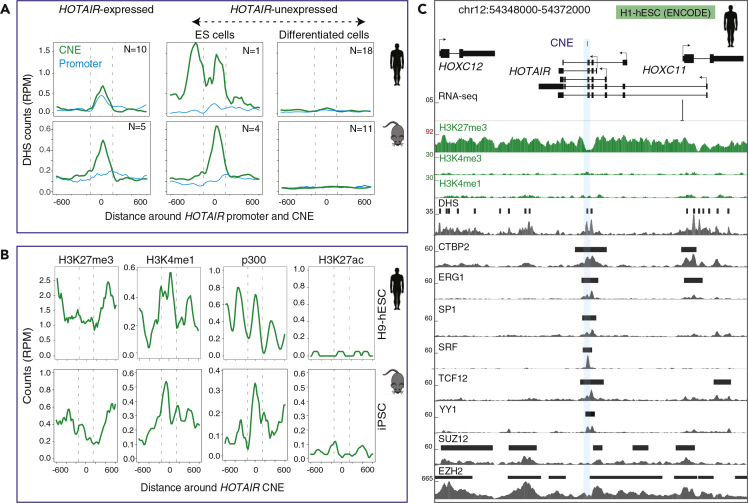
The *HOTAIR* CNE Represents a Poised Enhancer in *HOTAIR*-Nonexpressing Stem Cells in Human and Mouse (A) Average DNase I hypersensitive site (DHS) signals around *HOTAIR* CNE in *HOTAIR*-expressing and *HOTAIR*-nonexpressing cells (embryonic stem cells and differentiated cells). The y axis is normalized DHS coverage in reads per million (RPM). “N” denotes the number of cell lines. (B) The distribution of H3K4me1, H3K27me3, H3K27ac, and p300 signals around *HOTAIR* CNE in H9-hESC (human) and iPSC (mouse) cell lines. The y axis is normalized coverage in reads per million (RPM). (C) A genome browser view with transcription factors, DHS, histone modifications, and RNA-seq tracks from H1-hESC cell line. *HOTAIR* is not expressed in H1-hESC (as shown by lack of RNA-seq reads) and marked by broad H3K27me3 peak. H3K27me3 signal is depleted around CNE, reflecting a nucleosome-depleted region and bound by multiple transcription factors.

To understand whether chromatin states of CNEs resemble that of enhancers (Andersson et al., 2014, Pundhir et al., 2016, Roadmap Epigenomics Consortium et al., 2015), we selected 29 cell lines (Table S6) from Roadmap Epigenome project. Based on the expression levels of *HOTAIR* (see Methods), cell lines were classified into HOTAIR-expressing (N = 10) and HOTAIR-non-expressing (N = 19) groups (Figure S5A). The H1-hESC cell line is unique as it is enriched for H3K27me3 and DNase hypersensitive sites (DHSs) (Figure S5B), thus we separately analyzed H1-hESC cells from remaining *HOTAIR*-non-expressing cells. The *HOTAIR* CNE has open chromatin in HOTAIR-expressing cells and HOTAIR-non-expressing stem cells and closed chromatin in HOTAIR-non-expressing differentiated cells in both human and mouse (Figure 4A and Table S6). We observed a similar chromatin state around HOXD CNE (Figure S5C). The chromatin state of CNE is dynamically regulated during reprogramming of mouse embryonic fibroblasts to induced pluripotent stem cells (iPSCs) (Chronis et al., 2017) where the CNE has closed chromatin in mouse embryonic fibroblasts and open chromatin in iPSC (Figure S5D). In addition, enrichment of H3K4me1, H3K27me3, and p300 signals at the CNE in human H9-hESC and mouse iPSC (Figure 4B) provides evidence that the *HOTAIR* CNE represents an embryonic stem cell-specific poised enhancer (Rada-Iglesias et al., 2011). This is further supported by enrichment of enhancer-associated transcription factors (Sigova et al., 2015), such as CTBP2, CHD1, SP1, and YY1 that are exclusively enriched at CNE in H1-hESC (Figure 4C and Table S7). However, p300, H3K4me1, and bimodal H3K27me3 peaks were not enriched around HOXD CNE in hESC (Figures S5E–S5G). As HOXD CNE overlaps with the promoter of *ncHOXD11* (Figures 2A and S5G), genomic analyses of chromatin states will be unable to distinguish a putative enhancer from an overlapping promoter. Collectively, these data suggest that *HOTAIR* CNE resembles a poised enhancer in stem cells in both human and mouse.

### *HOTAIR* CNE Represents an Active Enhancer RNA

**Figure 5:**
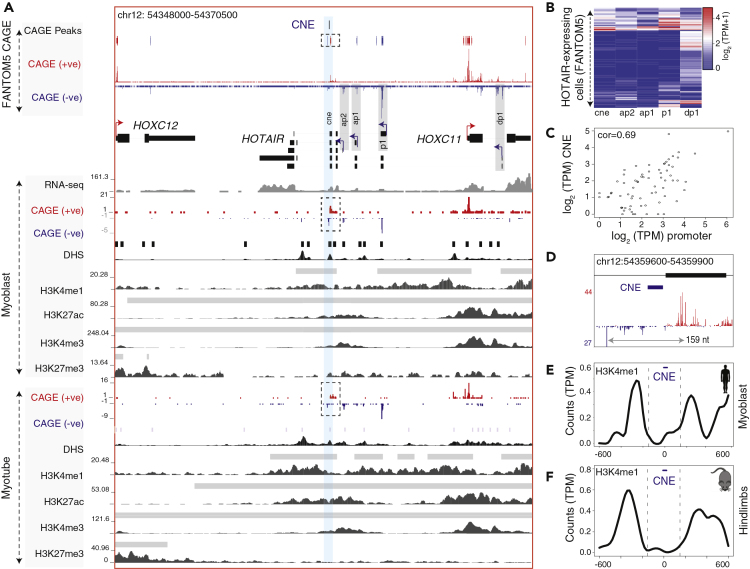
The CNE Represents an Active Enhancer RNA in *HOTAIR*-Expressing Cells (A) A genome browser view with FANTOM5 CAGE tags (combined tracks) and individual tracks on myoblast and myotube along with RNA-seq and histone modifications. Horizontal bars above histone and DHS tracks are annotated peaks. CAGE tags on forward and reverse strand are represented by blue and red, respectively. Bidirectional CAGE tags flanking the CNE are shown in dashed rectangular boxes. Bidirectional CAGE tags overlap with H3K4me1 and H3K27ac peaks in myoblast and myotube. (B) Expression levels of the *HOTAIR* CNE and alternative promoters across FANTOM5 samples. (C) Correlation of the expression levels of the CNE with that of *HOTAIR* promoter. (D) Bidirectional transcription (from myoblast and myotube differentiation time points) around the CNE roughly represents the length of a nucleosome. (E and F) Bimodal H3K4me1 peaks flank the CNE in human myoblast (E) and embryonic day 10.5 hindlimbs in mouse (F).

To determine whether the CNE represents an active enhancer in HOTAIR-expressing cells, we analyzed FANTOM5 CAGE data (FANTOM Consortium and the RIKEN PMI and CLST (DGT) et al., 2014) to identify unstable bidirectional transcription, a hallmark of active enhancer RNA (eRNA) (Andersson et al., 2014). We observed bidirectional transcription flanking the CNE (Figure 5A; dashed rectangular box), providing evidence of an active eRNA. Importantly, transcription of *HOTAIR* primary and alternative promoters is generally co-expressed with bidirectional transcription around the CNE (Figure 5B and Table S8). This is exemplified during myoblast to myotube differentiation (Figure S6A), which suggests coregulation. The expression of CNE is positively correlated with expression from the *HOTAIR* promoter and alternative promoters (Figure 5C), with the exception of the distal promoter (labeled as dp1) (Figure S6B). Negative correlation of the distal promoter is mostly due to a majority of samples in which only the distal promoter is expressed (Figure 5B). The distance between bidirectional transcription start sites flanking the CNE is about the length of one nucleosome (Figure 5D), which is conserved across vertebrates (Figure S1E), and suggests that the CNE shares an evolutionary conserved typical enhancer structure. On the contrary, no evidence suggests bidirectional transcription around the HOXD CNE, as it overlaps with the promoter region of *ncHOXD11* (Figure 2C).

Next, we focused on myoblast and myotube cell types, for which RNA-seq, histone modifications, and DHS data are available as complements to CAGE tags. The RNA-seq reads mapped on introns and across intron/exon boundary around the CNE (Figure 5A), thus providing evidence for an intron-retained transcript. Furthermore, DHS, H3K4me1, and H3K27ac peaks are enriched around the CNE (Figure 5A) in myoblast and myotube, providing additional evidence for an active enhancer. The observed bimodal H3K4me1 peaks around CNE are a characteristic feature of active enhancers (Figure 5E). However, in mouse, relevant tissues and stages wherein *Hotair* is expressed (Amandio et al., 2016, Li et al., 2013, Schorderet and Duboule, 2011) were not included in FANTOM5 samples (Figure S6C) and lack CAGE tags around the CNE. However, H3K4me1 and H3K27ac are enriched in mouse embryonic day 10.5 hindlimbs (Andrey et al., 2017) (Figures 5F and S6D) around the CNE. Thus, we showed that *HOTAIR* CNE resembles an active enhancer in *HOTAIR*-expressed cells, in both human and mouse. Importantly, transcription of the *HOTAIR* promoter is tightly linked to enhancer activity of the CNE, suggesting that its transcription might be a contributor to the purifying selection acting on the CNE and further provides support to the notion that the CNE acts as a regulator in *cis* as previously proposed (Amandio et al., 2016).

### *HOTAIR* Expression Highlights Simultaneous Regulation of Known Target Genes in *cis* and *trans*

**Figure 6:**
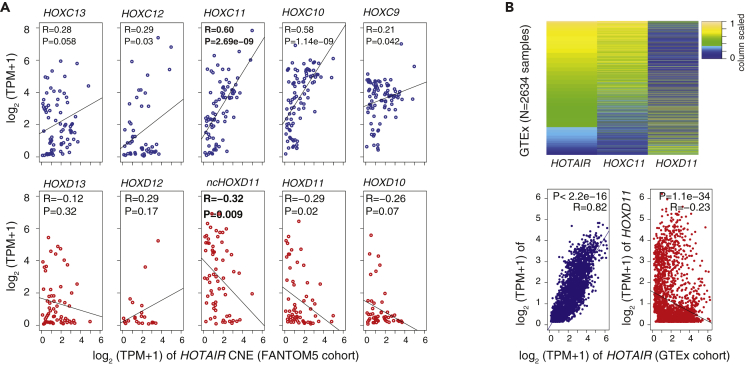
Coregulation of *HOTAIR* with Validated Target Genes *HOXC11* and *HOXD11* (A) Correlation of expression levels of *HOTAIR* CNE with HOXC and HOXD cluster posterior genes across FANTOM5 cell types. The x axis represents expression level of *HOTAIR* CNE, and the y axis represents the expression levels of HOXC and HOXD cluster genes. Expression level is measured as tags per million (TPM). The expression levels of *HOXC11* have the highest positive correlation, and those of the *HOXD11* coding and noncoding have the highest negative correlation. (B) Heatmap and correlation of expression levels of *HOTAIR* with *HOXC11* and *HOXD11* genes across GTEx cohort.

As we showed that transcribed CNEs exhibit sequence complementarity in transcript orientation, we sought to understand whether *HOTAIR* can simultaneously regulate genes in *cis* and *trans* mediated via the CNE. We analyzed transcription levels of the *HOTAIR* enhancer with HOX clusters genes across 694 cell types from FANTOM5. The *HOTAIR* was expressed in 104 cell types (Figure S7A), and HOX genes were positively correlated with other genes in the cluster (Figure S7B). The expression of *HOTAIR* CNE with HOXC/D clusters posterior genes on 104 cell types revealed positive correlation with HOXC cluster genes and a trend toward negative correlation with HOXD cluster genes (Figure S7C). To ensure that the correlations were not driven by missing expression of HOX genes, we reanalyzed data by including only those cell types wherein both *HOTAIR* and HOX genes are coexpressed. We observed similar correlations wherein HOXC cluster genes are positively correlated and HOXD cluster genes are negatively correlated (Figure 6A). Strikingly, *HOXC11* is the most positively correlated (R = 0.60; p value: 2.6 × 10^−9^) and *ncHOXD11* (R = −0.32; p value: 0.009) and coding transcripts (R = −0.29; p value: 0.02) are the most negatively correlated, both of which are previously reported target genes (Amandio et al., 2016, Li et al., 2013, Rinn et al., 2007). This observation was further validated in 2,436 tissue samples from GTEx and 605 patients with breast cancer (see Methods) from The Cancer Genome Atlas (Pereira et al., 2016) where *HOXC11* had the most significant positive correlation and *HOXD11* had the most significant negative correlation (Figures 6B and S7D).

Therefore, we propose a model to explain the observed correlation between *HOTAIR* expression and that of *HOXC11* and *HOXD11*, a dual regulatory mechanism mediated via the CNE sequence. The positive correlation between *HOTAIR* and *HOXC11* might be mediated via an active eRNA, the act of transcription of *HOTAIR,* or the combination of both. We observed positive correlation between *ncHOXD11* promoter encoding HOXD CNE and *HOXD11* coding gene. Transcriptional activity of the CNE is coupled to *HOTAIR* transcription, suggesting that a key function of the *HOTAIR* transcript could be to provide active transcription for the CNE. Paralogous CNEs embedded in intron-retained *HOTAIR* and *ncHOXD11* transcripts have retained sequence complementarity in transcript orientation that might facilitate hybridization between two RNA transcripts. This hybridization between two RNA transcripts downregulates *HOTAIR* target gene expression. Thus, we propose that *HOTAIR* can have dual regulatory roles in *cis* and *trans,* which is likely mediated by the CNE paralog sequence.

## Discussion

We have identified and characterized a 32-nucleotide CNE as the ancestral sequence that probably originated in ancestral HoxC/D cluster, where the second round of WGD gave rise to one copy in the *HOTAIR* locus and another copy in the *ncHOXD11* locus. The paralogous CNEs are only 32 nucleotides, whereas the conserved sequence flanking the *HOTAIR* CNE is much longer (Figure S1E) and coincides with a region of eRNA, suggesting an ancestral sequence within the *HOTAIR* locus. The remainder of the *HOTAIR* sequence has limited homology in vertebrates (Figures S1B and S1E), which evolved rapidly in mammalian lineage (He et al., 2011). This could be indicative of the *HOTAIR* locus originating from the CNE and evolution favoring the development of its sequence, likely expanding its functionality. Although thousands of CNEs are annotated, only a small minority of them have retained a duplicated copy (McEwen et al., 2006). As such, retention of both copies of *HOTAIR* CNE had not been reported before. To the best of our knowledge, this is the first instance of reported paralogous CNEs that underwent compensatory mutation and have retained sequence complementarity in their transcribed directionality (Figures 3A and 3B).

Many of the experimentally tested CNEs are validated enhancers (Nobrega et al., 2003, Pennacchio et al., 2006, Woolfe et al., 2005). Genome-wide transcriptomic and epigenomic analyses revealed that enhancers are characterized by distinct transcription and chromatin states (reviewed in Li et al., 2016b), and we used these features to define whether CNEs are enhancers. The *HOTAIR* CNE region is marked by open chromatin that is flanked by enriched H3K4me1 and H3K27ac peaks along with bidirectional transcription, which collectively meets all characteristic features of an active enhancer. On the other hand, our genomic analyses did not reveal any enhancer features on HOXD CNE, likely because it overlaps with the *ncHOXD11* promoter region (Figures 2C and 2D), and it is therefore difficult to entangle overlapping signals. However, the sequence overlapping Hoxd CNE drives expression in a proximal posterior part of the developing forelimbs in mouse (Beckers et al., 1996). Recent findings suggest that some promoters have dual functions as enhancers and influence the expression of a neighboring gene in *cis* (Engreitz et al., 2016, Paralkar et al., 2016, Yin et al., 2015). Thus, it is plausible that the *ncHOXD11* promoter overlapping HOXD CNE has enhancer function and regulates *HOXD11* gene in *cis*.

Multiple enhancers with similar activity provide an effective buffer to prevent deleterious phenotypic consequences upon loss of individual enhancers (Osterwalder et al., 2018). As the *HOTAIR* CNE has a paralogous copy, how this might affect *HOTAIR* regulation needs further consideration. Deletion of a sequence overlapping Hoxd CNE revealed no phenotype *in vivo* (Beckers and Duboule, 1998), which was different from *in vitro* (Beckers et al., 1996). It was speculated that the difference(s) might be due to other phenotypes that were undetected or might have a redundant copy that masked the effect. In fact, we now have identified that the probed sequence has a paralogous copy in the *Hotair* locus that might have masked the effect *in vivo*. Thus, whether paralogous CNEs have redundant functions, such that deletion of one CNE might be compensated by the other, remains unclear. Putting this in the context of deletion of the *Hotair* locus *in vivo* (Amandio et al., 2016, Li et al., 2013), it remains unknown whether the effects of *Hotair* CNE deletion are compensated for, to a certain extent, by paralogous Hoxd CNE.

Transcriptional activity of CNEs is coupled to *HOTAIR* and *ncHOXD11* transcription, suggesting that a key function of these transcripts is to provide active transcription for the CNEs. With respect to transcript orientation, paralogous CNEs exhibit sequence complementarity, which raises the potential for this hybridization principle based on *trans* function. This is supported by the observed hybridization *in vitro* (Figures 3C and 3D) and needs future experiments to confirm *in vivo*. Transcription of *HOTAIR* CNE is positively correlated with *HOXC11* (Figure 6), and transcription of *ncHOXD11* is positively correlated with *HOXD11*. Simultaneously, the transcription of *HOTAIR* CNE and *ncHOXD11* are negatively correlated (Figure 6), which is likely mediated via sequence complementarity between CNEs.

In summary, our analyses suggest that *HOTAIR* could regulate both HoxC and HoxD cluster genes simultaneously and provide a unifying model of *HOTAIR* regulation that should clarify ongoing controversies (Amandio et al., 2016, Li et al., 2013, Portoso et al., 2017, Rinn et al., 2007, Schorderet and Duboule, 2011). Our work highlights how an lncRNA locus could possibly function at the DNA and RNA levels to regulate genes both in *cis* and *trans*. Unraveling such lncRNAs and determining/validating mechanisms through which they function at the DNA and/or RNA levels is an ongoing challenge. We propose that such integrative analyses bridging evolutionary genomics and comparative transcriptomics/epigenomics could prove a powerful tool for better understanding of lncRNA-dependent regulation processes.

### Limitations of the Study

Our conclusion is based on analyses of large-scale genomics data, thus future work is needed to validate the predicted models *in vivo*. We showed hybridization between paralogous CNEs *in vitro*, which needs to be validated *in vivo*. Furthermore, targeted experiments are required to understand how specific deletion of individual CNE(s) along with simultaneous deletion of both CNEs alters *HOTAIR*-dependent regulation in *cis* and *trans*.

## Methods

All methods can be found in the accompanying Transparent Methods supplemental file.

## Data and Code Availability

Data used in the study were downloaded from ENCODE, mouse ENCODE, GTEx, TCGA, FANTOM5, NIH Roadmap Epigenome project, and additional publicly available datasets mentioned in the methods. All custom code is available upon request.

## Supplemental Information

Document S1. Transparent Methods and Figures S1–S7

Table S1. Coordinates of CNE in HoxC and HoxD Clusters across Vertebrates, Related to Figure 1

Table S2. Summary of Annotation of HoxC Cluster Genes in Aves and Teleosts, Related to Figure 1

Table S3. Read Counts on *HOTAIR* Introns and Exons across ENCODE Cell Types, Related to Figure 2

Table S4. Sequences around CNEs to Design RNA-Oligo, Related to Figure 3

Table S5. Concentration of Titrated RNA-Oligo, Related to Figure 3

Table S6. Roadmap Epigenome Cell Lines Classified into Three Groups Based on *HOTAIR* Expression, Related to Figure 4

Table S7. Enrichment of Transcription Factors (TFs) Bound on *HOTAIR* CNE and Promoter, Related to Figure 4

Table S8. Expression Levels of *HOTAIR* CNE, Promoter, and Distal Promoter across FANTOM5 Samples, Related to Figure 5
