# Supplementary material for: Ancestrally Duplicated Conserved Noncoding Element Suggests Dual Regulatory Roles of HOTAIR in cis and trans
Source: iScience. 2020 Mar 25;23(4):101008. doi: 10.1016/j.isci.2020.101008 (PMC7139118; doi:10.1016/j.isci.2020.101008)
Supplement: Document S1. Transparent Methods and Figures S1–S7 [file mmc1.pdf]

iScience, Volume *23*

Supplemental Information

Ancestrally Duplicated Conserved Noncoding Element Suggests Dual Regulatory Roles of HOTAIR in *cis* and *trans*

Chirag Nepal, Andrzej Taranta, Yavor Hadzhiev, Sachin Pundhir, Piotr Mydel, Boris Lenhard, Ferenc Müller, and Jesper B. Andersen

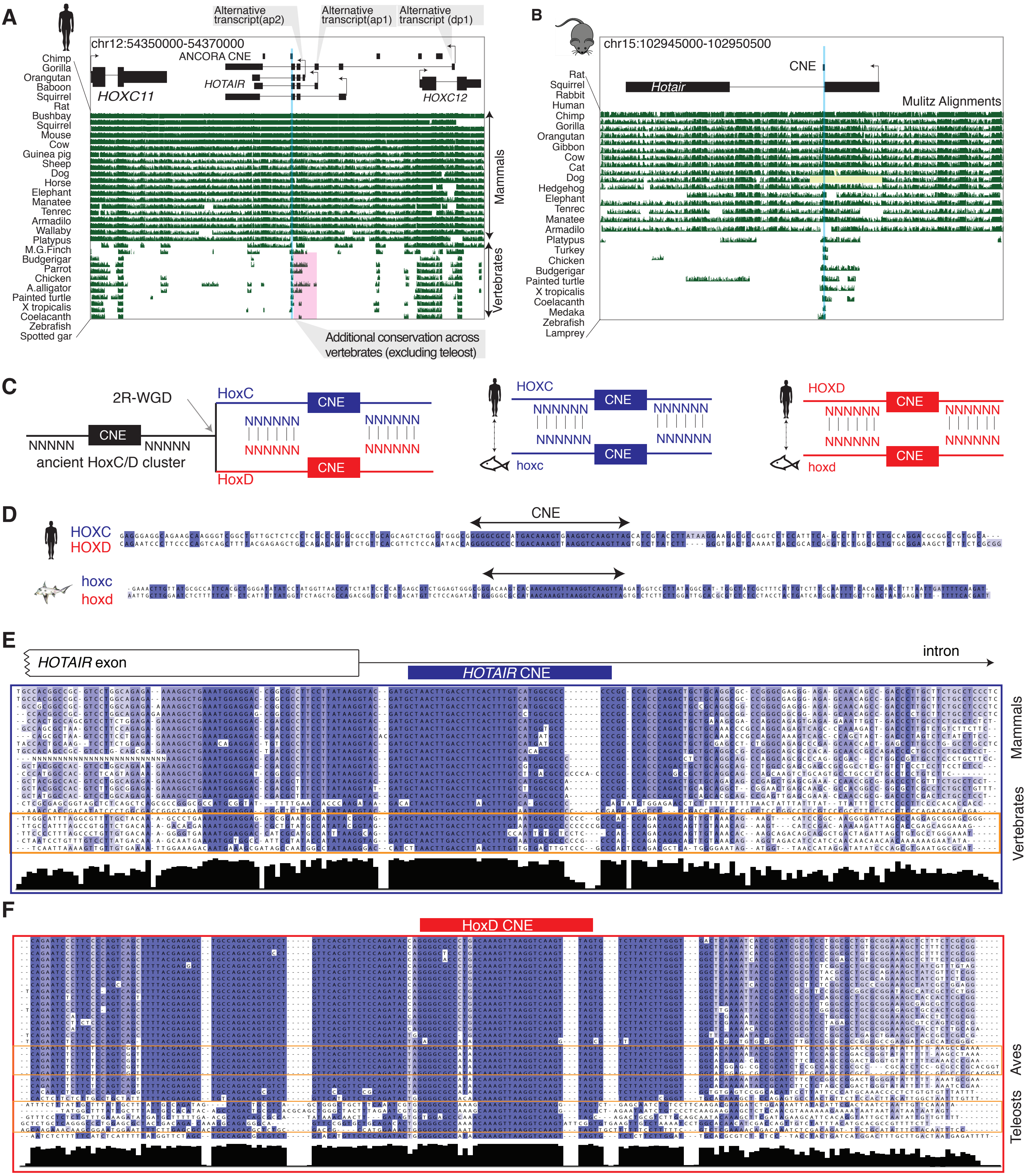

Figure S1. Sequence alignment of *HOTAIR*. Related to Figure 1. (**A-B**) A genome browser view of *HOTAIR* in human (A) and mouse (B) show sequence alignments across vertebrates. Conserved noncoding elements (CNEs) track from ANCORA browser is shown as on top. CNE that is conserved across all mammals and vertebrates is highlighted. (**C**) Schematic representation to show the origin of HoxC and HoxD cluster from the ancestral HoxC/D cluster after second round of whole genome duplication (2R-WGD). Schematic representations of HoxC and HoxD clusters separately across vertebrates. (**D**) Alignment of sequences flanking paralogous CNEs in human and elephant shark show little conservation despite both sequences being duplicated from the same ancestral sequences. (**E-F**) Alignment of sequences flanking *HOTAIR* CNE (**E**) and HOXD CNE (**F**). Species are aligned in the same order as in A.

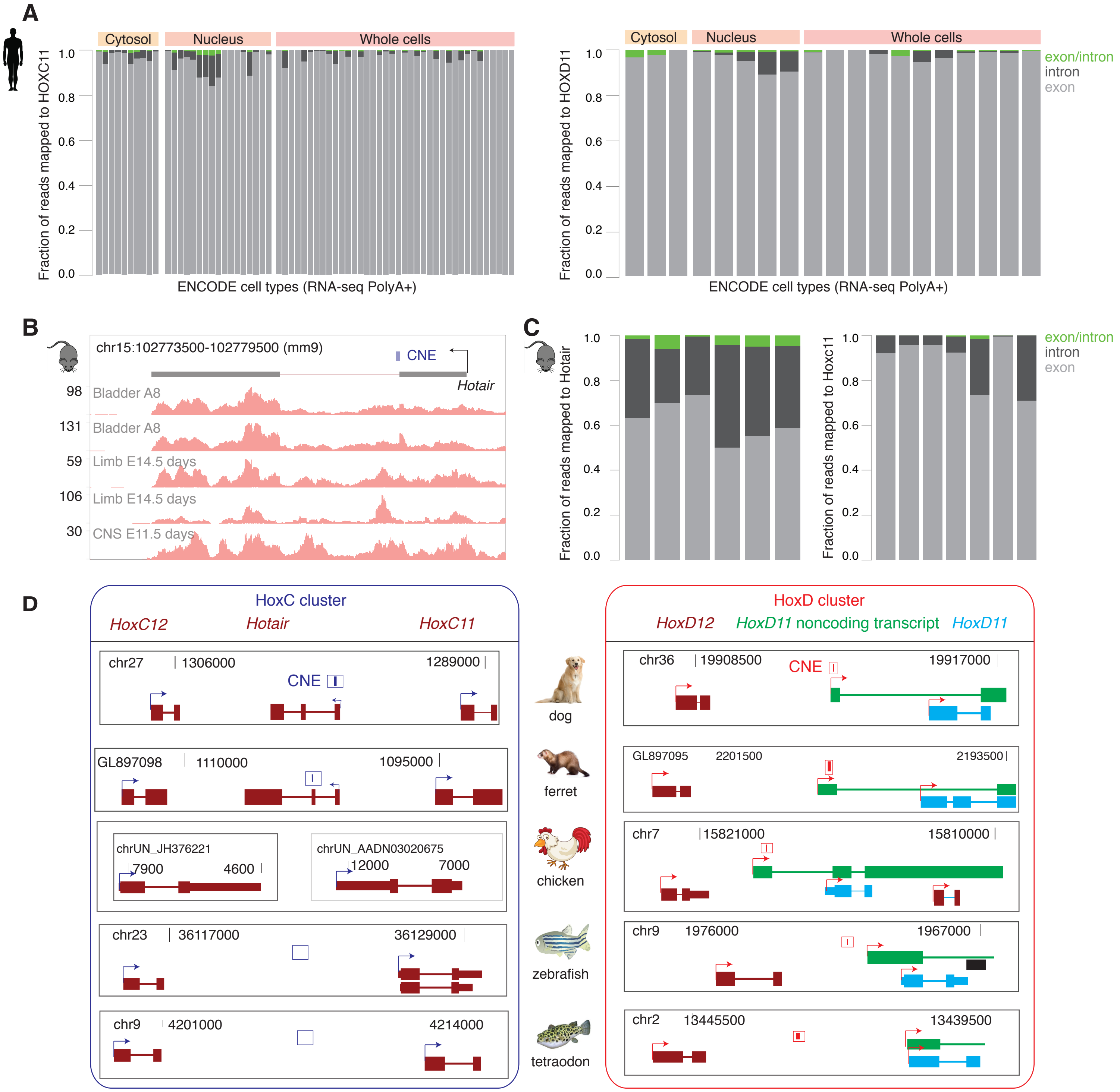

Figure S2. Paralogous CNEs are embedded in mature noncoding transcrips. Related to Figure 2. (**A**) Distribution of reads mapped to HOXC11 and HOXD11 exon, intron and overalpping exon/intron junctions across multiple cell types. Cells types are ordered based on increasing number of mapped reads. (**B**) A geneome browser view of Hotair transcript along with RNA-seq coverage tracks across different cell types from mouse ENCODE. (**C**) Distribution of reads mapped to mouse Hotair and Hoxc11 across multiple cell types. Cells types are ordered based on increasing number of mapped reads. (**D**) Evidence of *HOTAIR* and *HoxD11* noncoding transcript across multiple species. The CNEs are represented by rectangular blue and red bar in HoxC and HoxD cluster respectively. The *hoxc11* and *hoxc12* genes are assembled in different contigs in chicken, and homolog of *HOTAIR* CNE is undetected because the intergenic region between *hoxc11* and *hoxc12* is not assembled. Zebrafish *hoxc11* and *hoxc12* is assembled but lacks the CNE. *HoxD11* noncoding transcript is detected across tetrapods but not in teleosts (zebrafish and tetraodon).

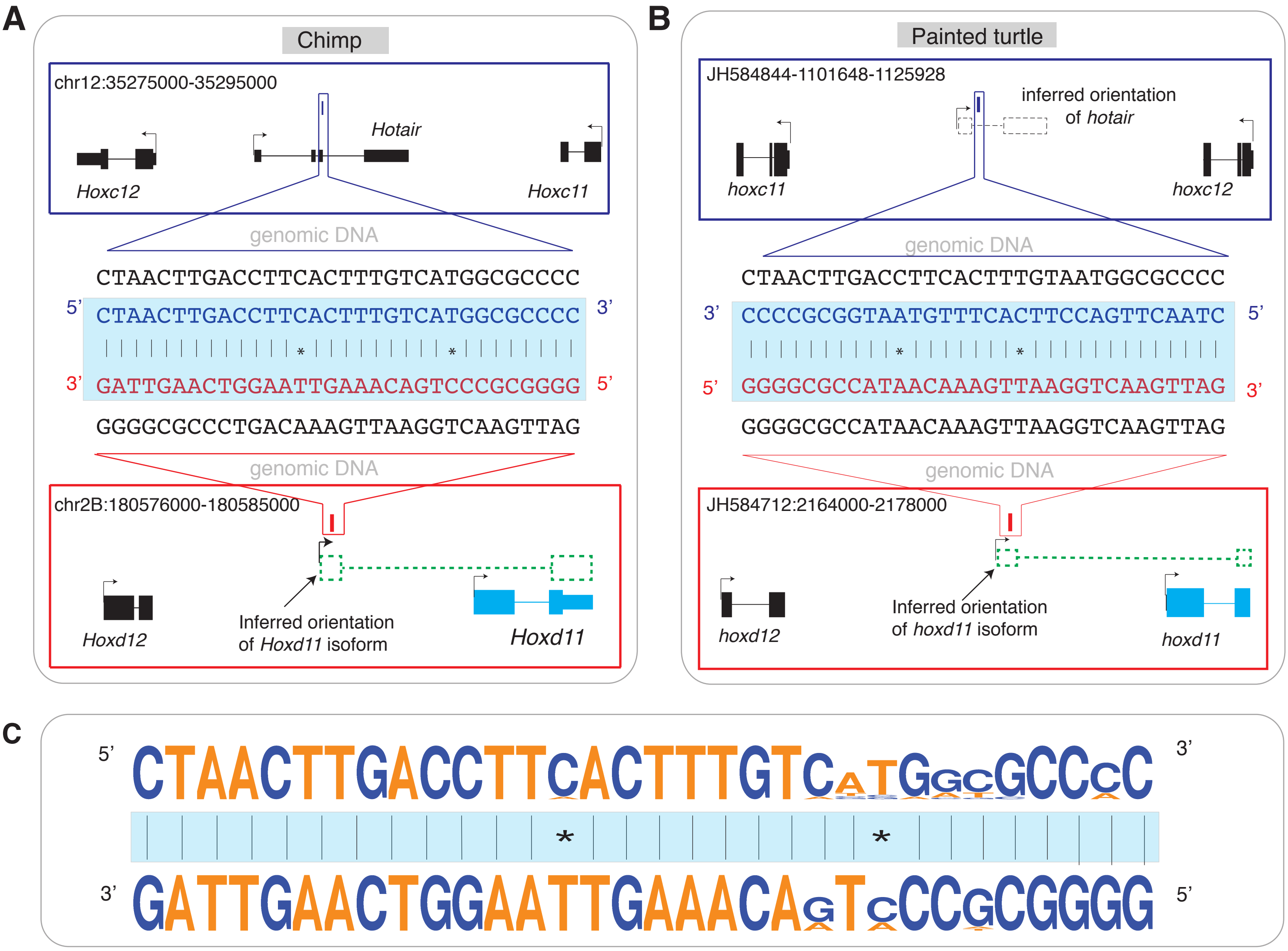

Figure S3. Paralogous CNEs exhibit sequence complementarity with respect to transcription directionality of *HOTAIR* and *HoxD11* noncoding transcript (*ncHOXD11*). Related to Figure 3. (**A-B**) Schematic representation to depict the inferred orientation of missing transcripts in chimp and painted turtle. *HOTAIR* is antisense to *HoxC11* gene, so the same convention was used to infer the orientation of *HOTAIR*. The *ncHOXD11* is an alternative splice variant of *HoxD11* coding gene across multiple species; thus, the same convention was used. Expected transcripts are represented by dashed rectangular boxes and lines. Arrows indicate directionality of transcript. The CNE sequences are zoomed in and shown as genomic DNA and transcribed RNA. Paralogogous CNEs exhibit sequence complementarity when aligned in 5' to 3' orientation. (**C**) Sequence logos of *HOTAIR* CNE and HoxD CNE show paralogous CNEs exhibit sequence complementarity in transcribed orientation.

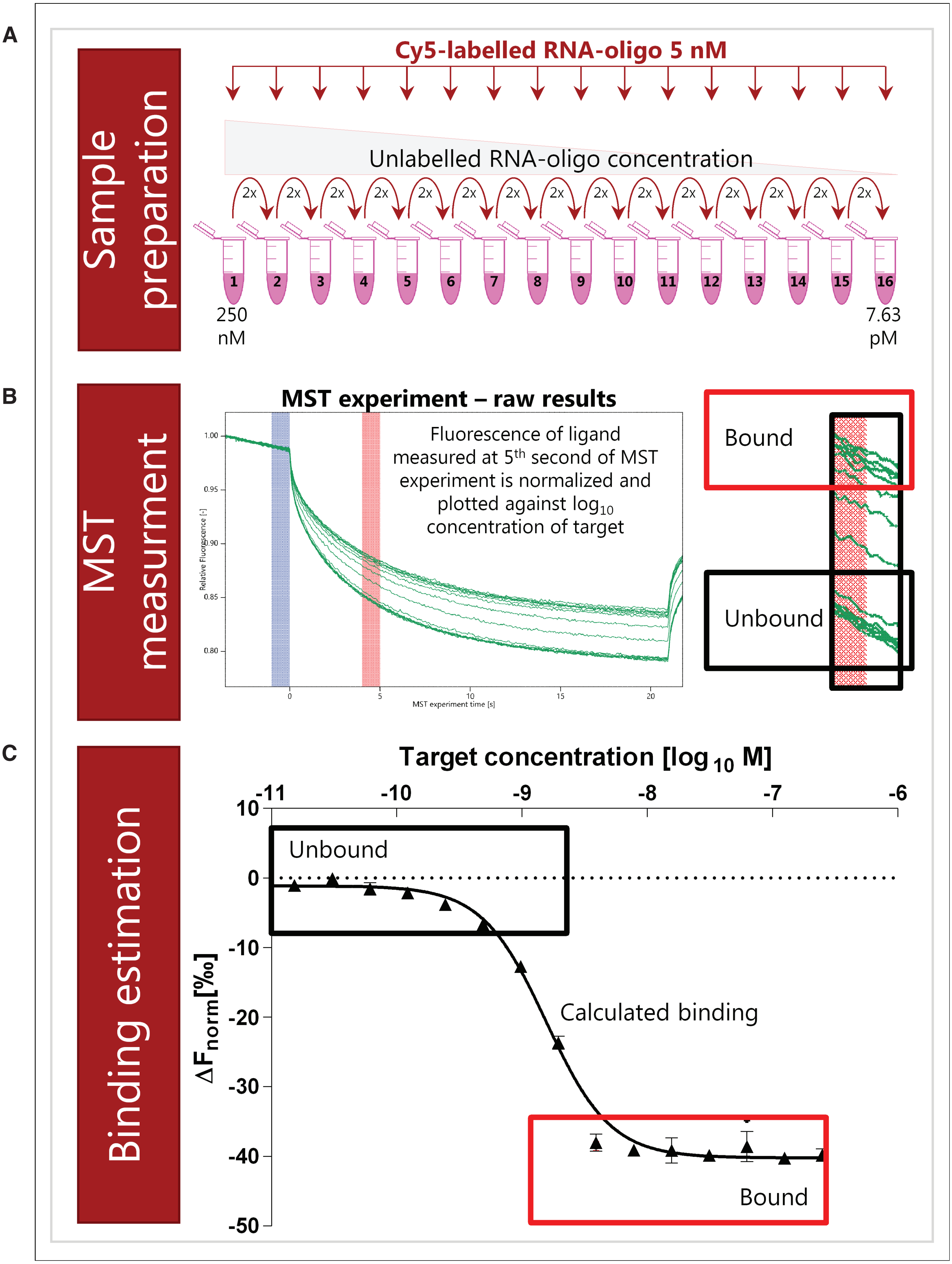

Figure S4. A schematic workflow to describe samples preparation for miscroscale thermophoresis (MST). Related to Figure 3. (**A**) Two-fold dilutions of unlabeled RNA-oligo were prepared starting at 250nM concentration. Labelled RNA-oligo was kept constant at 5nM. (**B**) An illustrative example of raw experimental data. Fluorescent of labelled RNA-oligo was measured at 5th second of the MST experiment. (**C**) Raw data were normalized as ΔF_{norm} [‰] and plotted against $\log_{10}$ concentration of titrated RNA-oligo.

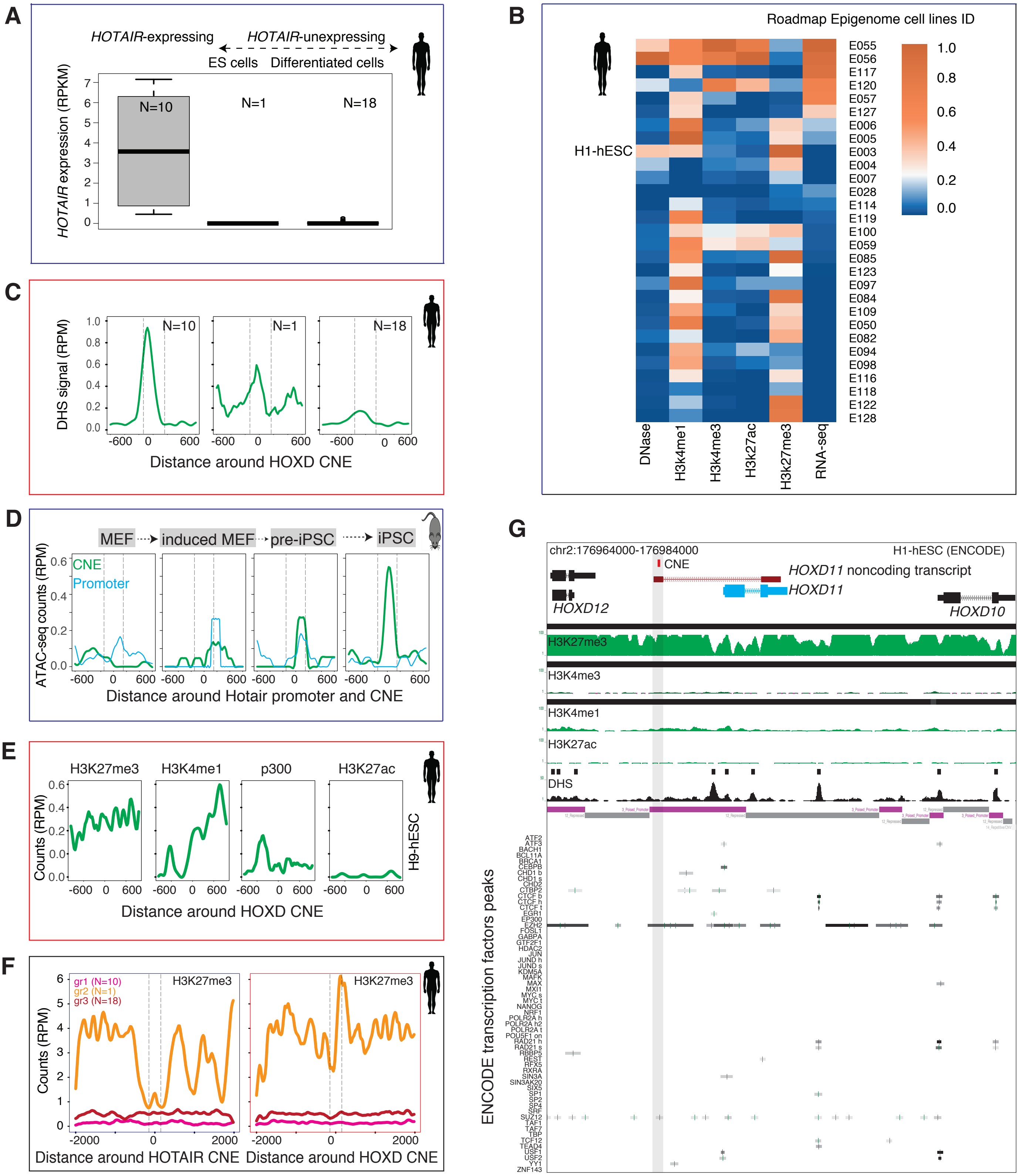

Figure S5. Transcription and chromatin environment of parlogous CNEs. Related to Figure 4. (**A**) Expression levels of *HOTAIR* in twenty-nine cell lines from Roadmap Epigenome data. A threshold of 0.5 RPKM (reads per kilobase per million mapped reads) was used to separate HOTAIR-expressing cell lines. HOTAIR-unexpressing cell lines were further separated into stem cells (N=1) and terminally differentiated cells (N=18). (**B**) Heatmap shows the normalized read counts in 250 nucleotides flanking *HOTAIR* CNE across 29 cell lines. (**C**) DHS signals around the HOXD CNE across three groups. Y-axis represents normalized counts in reads per million (RPM). (**D**) Dynamics regulation of chromatin state around mouse *Hotair* CNE during reprogramming of mouse embryonic fibroblast to iPSC. (**E**) Distribution of H3K4me1, H3K27me3, H3K27ac and p300 signals around human HOXD CNE in H9-hESC cell line. (**F**) Pattern of H3K27me3 marks around paralogous CNEs across three groups. (**G**) A genome browser view around HOXD CNE shows enrichment of multiple signals (transcription factors, DHS, histone modifications, chromHMM marks) in H1-hESC cell line.

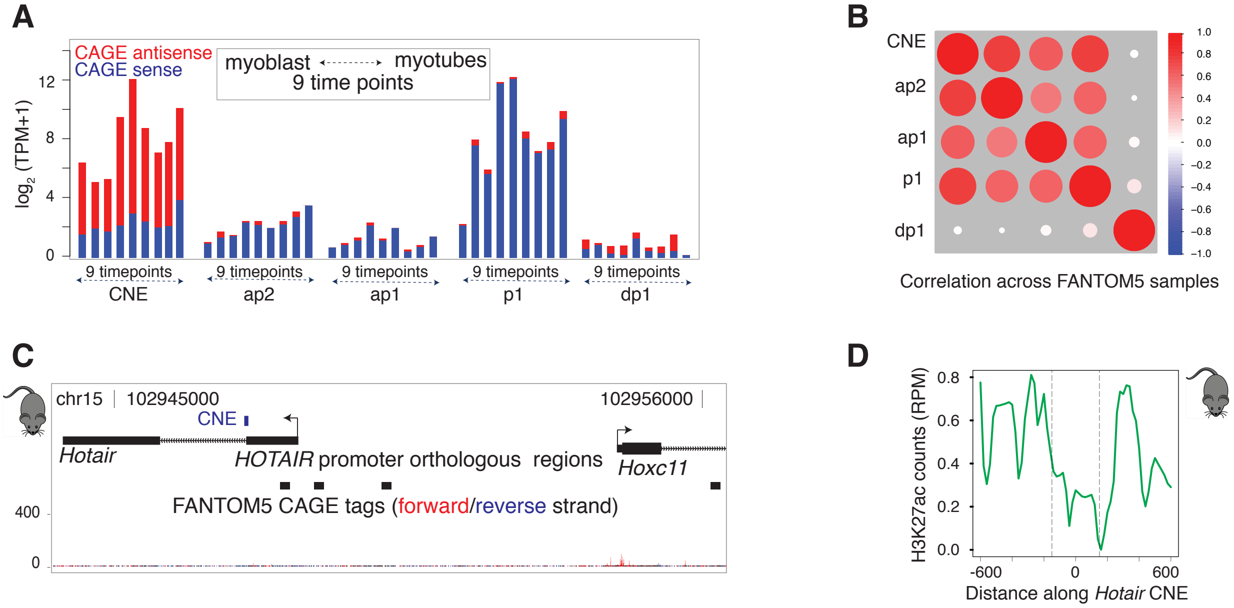

Figure S6. Transcriptional dynamics of *HOTAIR* promoters. Related to Figure 5. (**A**) Expression levels of *HOTAIR* promoter (p1), alternative promoters (ap1, ap2), distal promtoer (dp1) and CNE across nine different time points during differentiation from myoblast to myotube. (**B**) Expression correlation of *HOTAIR* promoter, alternative promoters and CNE across FANTOM5 samples are positively correlated except for distal promoter (dp1). (**C**) Distribution of CAGE tags around mouse *Hotair* locus in FANTOM5 samples. No significant CAGE tags are detected as it lacks tissues (embryonic hindlimbs, genital tubercle and a piece of trunk corresponding to sacro-caudal region) where *Hotair* is expressed. Orthologs of promoter region of *HOTAIR* are aligned to mouse. (**D**) *Hotair* CNE is flanked by bidirectional H3K27ac on mouse hindlimbs (E10.5 days).

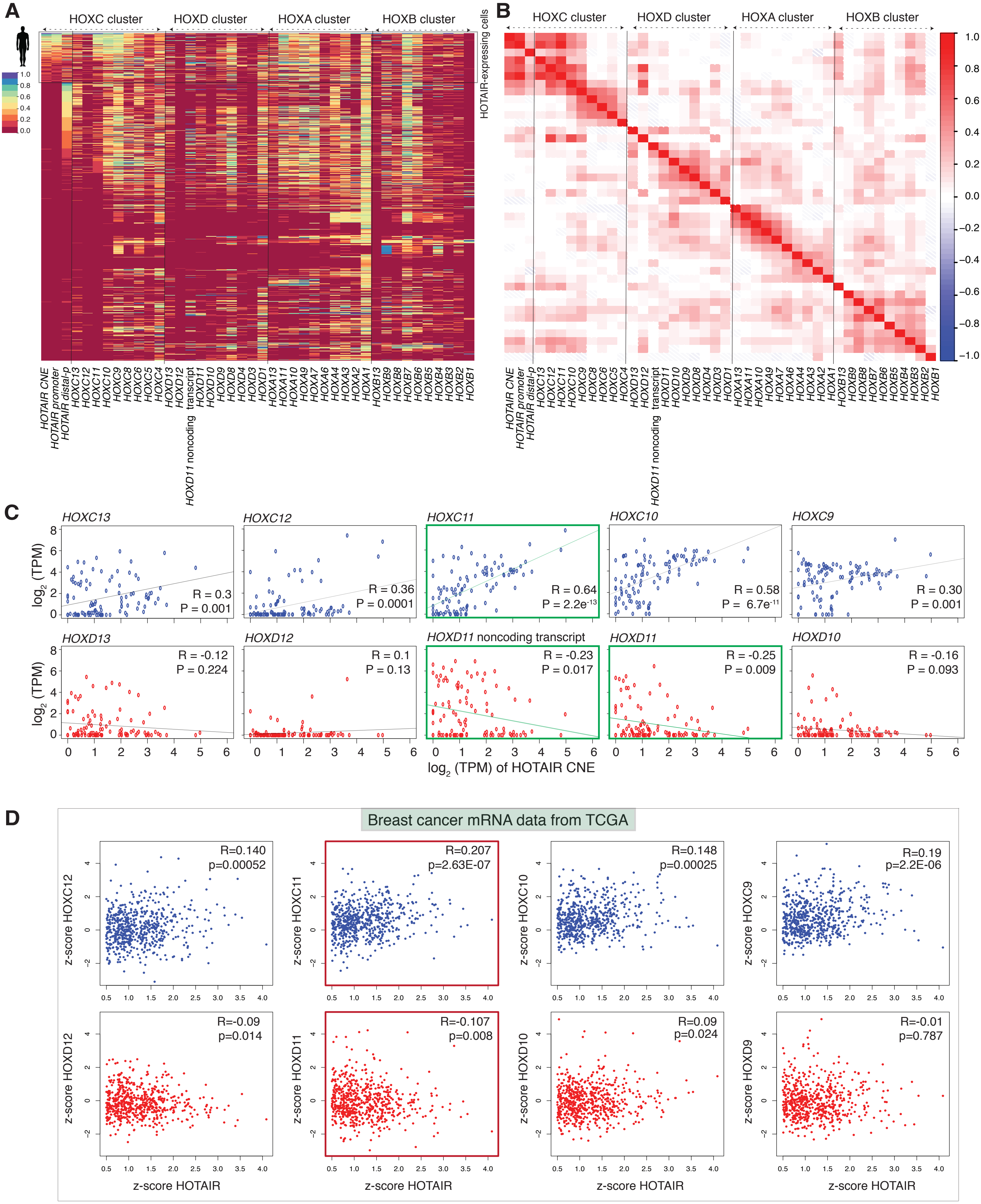

Figure S7. Co-expression analysis of *HOTAIR* with genes from four HOX clusters. Related to Figure 6. (**A**) Heatmap shows the expression level of *HOTAIR* and four HOX cluster genes across FANTOM5 samples. Cell types are sorted based on the expression levels of *HOTAIR* CNE. Expression levels are scaled between 0-1 across each column. (**B**) Correaltion of expression levels of *HOTAIR* with genes from four HOX clusters. (**C**) Correaltion of expression levels of *HOTAIR* CNE with HOXC and HOXD cluster posterior genes. Expression level is measured as tags per million (TPM). The most positively correalted gene (*HOXC11*) and most negatively correalted gene (*HOXD11* noncoding transcript) is highlighted in green border. **(D)** Correaltion of expression levels of *HOTAIR* with HOXC and HOXD cluster posterior genes on individual breast cancer patients.

Transparent Methods

Genome assemblies and gene annotations

Analyses on human and mouse were done in hg19 and mm9 genome version respectively. The genome assemblies of 37 species are listed in Table S1. Gene models were downloaded from UCSC (Speir et al., 2016). The conserved noncoding elements (CNEs) were downloaded from ANCORA (Engstrom et al., 2008).

Roadmap Epigenome data sets

Roadmap Epigenome data were downloaded from NIH Roadmap Epigenome browser (Roadmap Epigenomics et al., 2015). Annotated chromatin states were downloaded from 127 cell lines. Histone modifications (H3K4me1/3, H3K27ac/me3) and DNase I hypersensitive sites (DHSs) data were downloaded as mapped (tagAlign format) files. RNA-seq data for 57 cell lines were downloaded in the computed gene expression (RPKM) matrix (Roadmap Epigenomics et al., 2015). Only 29 cell lines that had all four (H3K4me1/3, H3K27ac/me3) histone modifications, DHS and RNA-seq were used for downstream analyses.

ENCODE and mouse ENCODE data sets

Histone modification and RNA-seq data from ENCODE were downloaded as mapped BAM files. ENCODE transcription factor ChIP-seq were downloaded as annotated peaks (Gerstein et al., 2012). Histone modifications (H3K4me1/3, H3K27ac/me3), DHS and RNA-seq data from mouse ENCODE were downloaded as mapped BAM files (Yue et al., 2014). Samples with replicates were merged into a single file. A threshold of 0.5 RPKM (reads per kilobase per million) was used as the cutoff expression to determine whether *HOTAIR* is expressed or not in the given RNA-seq samples.

FANTOM5 data sets

Human and mouse CAGE-seq data were downloaded from FANTOM5 (Arner et al., 2015; Consortium et al., 2014). Replicates were pooled into single file and resulting CAGE tags in each sample were quantified as tags per million (TPM). CAGE tags with the highest expression level were defined as the dominant transcription start site (TSS). CAGE based expression level was computed by summing all CAGE tags in the defined promoter region (300 bases upstream and downstream of TSS). To compare the expression correlation across four HOX clusters genes, we selected only that samples/cell types if any of HOX genes had a minimum expression level of 5 TPM, which resulted in a total of 694 cell types.

GTEx RNA-seq data sets

Mapped GTEx RNA-seq expression data (Consortium, 2013) for genes and transcripts were downloaded from GTEx_Analysis_2017-06-05_v8_RNASeQCv1.1.9_gene_tpm.gct.gz and GTEx_Analysis_2017-06-05_v8_RSEMv1.3.0_transcript_tpm.gct.gz respectively. These data contain 17382 samples from different tissues. For comparative analysis of expression levels of *HOXD11* coding and noncoding transcripts, we selected only those samples where both

transcripts had a minimum expression level of 0.1 TPM and additionally one of the transcripts had a minimum expression level of 0.5 TPM, which resulted in 1830 samples. For comparative analysis of expression levels of *HOTAIR*, *HOXC11* and *HOXD11* gene, we selected only those samples where all three transcripts had a minimum expression level of 0.1 TPM and additionally one of the transcripts had a minimum expression level of 0.5 TPM, which resulted in 2633 samples.

Data sets used from multiple studies

RNA-seq transcripts for multiple species were used from previous studies (Basu et al., 2016; Hezroni et al., 2015; Nepal et al., 2013). Raw data during reprogramming of mouse embryonic fibroblast to iPSC were download from GEO (GSE90894) (Chronis et al., 2017). Raw data for H3K27me3, H3K4me1, H3K27ac and p300 from H9-hESC cell lines were download from GEO (GSE24447) (Rada-Iglesias et al., 2011). Mouse embryonic (10.5 days) hind limb data were downloaded from GEO (GSE84793) (Andrey et al., 2017). Raw fastq reads were mapped using bowtie2 (Langmead and Salzberg, 2012). Only unique mapping reads were considered for downstream analysis. Breast cancer patients' mRNA (Illumina Human v3 microarray) data (Pereira et al., 2016) were downloaded from TCGA portal. Expression levels are measured in z-scores. We filtered samples where *HOTAIR* expression (<= 0.5) and were left with 605 patients.

Intron retention reads of *HOTAIR*

For intron retention analysis, we downloaded long RNA-seq data from ENCODE in human and mouse, in the form of mapped BAM files. For intron retention analysis, only polyA+ libraries were analyzed, and further classified into whole cell, nuclear fraction and cytosol fraction enriched libraries. To compute the ratio of intron and exon reads, we used gene annotation from RefSeq and computed the number of reads mapped to exons and introns. We only included samples if the total number of reads mapped to *HOTAIR* was higher than hundred. The sequence reads that were unspliced and overlapped the exon/intron junctions were counted separately from exonic and intronic reads.

Mapping of *HOTAIR* CNE across multiple species

The *HOTAIR* CNE sequences from both human and zebrafish was used as a query sequence. We used BLAST (blastall –p blastn –d –e 0.01 –m 8)(Altschul et al., 1997) to find homologous sequences against 37 species (Supplementary Table S1). Even at the permissive e-value cutoff of 0.01, only two homologous sequences were identified.

Annotation and directionality of *HOTAIR* and *HOXD11* noncoding transcripts overlapping CNEs

The *HOTAIR* transcript is annotated in multiple species (Hezroni et al., 2015; Speir et al., 2016), such as human, chimp, mouse, ferret and dog, and its orientation is antisense to *HoxC11* and *HoxC12* genes. Species lacking *HOTAIR* annotation, orientation of *HOTAIR*

CNE was assigned antisense to annotated HoxC cluster genes. In multiple species, such as human, chimp, mouse, ferret, dog and chicken, HoxD CNE is embedded within the exon of *ncHoxD11*, which is an alternative transcript of *HoxD11* coding gene. Thus, orientation of HoxD CNE was assigned similar to annotated *HoxD11* gene. Among teleosts fish, we analyzed RNA-seq transcripts in zebrafish (Hezroni et al., 2015; Nepal et al., 2013) and tetraodon (Basu et al., 2016), and did not identify *ncHoxD11*.

Software and tools

Multiple alignments were generated using ClustalW (Chenna et al., 2003) and Jalview (Waterhouse et al., 2009). Sequence logos were generated using WebLogo (Crooks et al., 2004). Data were visualized by uploading bigwig tracks on UCSC genome browser and images were downloaded. Bedtools (Quinlan and Hall, 2010), bash, perl and R scripts were used for data analysis.

Microscale thermophoresis experiment

The microscale thermophoresis (MST) is based on the phenomenon of molecule drift in temperature gradient (Asmari et al., 2018; Duhr and Braun, 2006a, b; Moon et al., 2018). In constant buffer conditions, thermophoresis depends on molecule size, charge and solvation entropy (hydration shell) which may change upon ligand binding. To measure the thermophoretic effect, the ligand is fluorescently labelled and kept at a constant concentration, whereas its interactor is titrated. Change in fluorescence emission at different ligand concentrations reflects an altered response based on the force of a temperature gradient. Plotting of fluorescent signal change against altered ligand concentration allow the calculation of Kd/EC_{50}.

Cy5-labelled or unlabelled RNA oligonucleotides (Supplementary Table S3) corresponding to CNEs and short flanks were used (TAG Copenhagen). To minimize potential influence of labelling on CNEs' interaction experiment was set up with mixtures of HOXD with labelled HOTAIR-Cy5, and HOXC with Cy5-HOXD. Fluorescent and regular RNA oligos dilutions were prepared in 1x MST buffer. In initial experiment MST buffer and MST buffer with addition of unspecific RNA was tested returning comparable results. Thus, data presented here were recorded on samples prepared in 1x MST buffer only. Unlabelled oligo was prepared as serial 2x dilutions in 15 µL volume accordingly to manufacturer recommendation. Fifteen µL of labelled 10 nM oligo was added to serial dilutions and mixed by pipetting. Final concentration of labelled RNA-oligo was 5nM and ligand was in range of 250 nM to 7.63 pM. Regular RNA-oligo corresponding to the labelled probe used in particular experimental setup was used as competitor. Details on samples and RNA-oligo types and concentration used are described (Supplementary Table S4). After short incubation, prepared mixtures were loaded into Standard Treated Capillaries and MST signal was measured on Monolith NT.115 (NanoTemper Technologies) with default settings (auto-detect LED and medium MST power).

Each experimental condition was run at least 3 times. Representative graphs of raw MST data are depicted on (Figure S4). For the analysis baseline corrected normalized fluoresce (ΔF_{Norm}) was used as recommended in MST software manual, and plotted against the log10 ligand concentration in GrapPad Prism 7 (GrapPad Software). The threshold values were extrapolated from sigmoidal fitting curve.

Supplemental References

Altschul, S.F., Madden, T.L., Schaffer, A.A., Zhang, J., Zhang, Z., Miller, W., and Lipman, D.J. (1997). Gapped BLAST and PSI-BLAST: a new generation of protein database search programs. Nucleic Acids Res *25*, 3389-3402.

Andrey, G., Schopflin, R., Jerkovic, I., Heinrich, V., Ibrahim, D.M., Paliou, C., Hochradel, M., Timmermann, B., Haas, S., Vingron, M.*, et al.* (2017). Characterization of hundreds of regulatory landscapes in developing limbs reveals two regimes of chromatin folding. Genome Res *27*, 223-233.

Arner, E., Daub, C.O., Vitting-Seerup, K., Andersson, R., Lilje, B., Drablos, F., Lennartsson, A., Ronnerblad, M., Hrydziuszko, O., Vitezic, M.*, et al.* (2015). Transcribed enhancers lead waves of coordinated transcription in transitioning mammalian cells. Science *347*, 1010-1014.

Asmari, M., Ratih, R., Alhazmi, H.A., and El Deeb, S. (2018). Thermophoresis for characterizing biomolecular interaction. Methods *146*, 107-119.

Basu, S., Hadzhiev, Y., Petrosino, G., Nepal, C., Gehrig, J., Armant, O., Ferg, M., Strahle, U., Sanges, R., and Muller, F. (2016). The Tetraodon nigroviridis reference transcriptome: developmental transition, length retention and microsynteny of long non-coding RNAs in a compact vertebrate genome. Sci Rep *6*, 33210.

Chenna, R., Sugawara, H., Koike, T., Lopez, R., Gibson, T.J., Higgins, D.G., and Thompson, J.D. (2003). Multiple sequence alignment with the Clustal series of programs. Nucleic Acids Res *31*, 3497-3500.

Chronis, C., Fiziev, P., Papp, B., Butz, S., Bonora, G., Sabri, S., Ernst, J., and Plath, K. (2017). Cooperative Binding of Transcription Factors Orchestrates Reprogramming. Cell *168*, 442-459 e420.

Consortium, F., the, R.P., Clst, Forrest, A.R., Kawaji, H., Rehli, M., Baillie, J.K., de Hoon, M.J., Haberle, V., Lassmann, T.*, et al.* (2014). A promoter-level mammalian expression atlas. Nature *507*, 462-470.

Consortium, G.T. (2013). The Genotype-Tissue Expression (GTEx) project. Nat Genet *45*, 580-585.

Crooks, G.E., Hon, G., Chandonia, J.M., and Brenner, S.E. (2004). WebLogo: a sequence logo generator. Genome Res *14*, 1188-1190.

Duhr, S., and Braun, D. (2006a). Optothermal molecule trapping by opposing fluid flow with thermophoretic drift. Phys Rev Lett *97*, 038103.

Duhr, S., and Braun, D. (2006b). Why molecules move along a temperature gradient. Proc Natl Acad Sci U S A *103*, 19678-19682.

Engstrom, P.G., Fredman, D., and Lenhard, B. (2008). Ancora: a web resource for exploring highly conserved noncoding elements and their association with developmental regulatory genes. Genome Biol *9*, R34.

Gerstein, M.B., Kundaje, A., Hariharan, M., Landt, S.G., Yan, K.K., Cheng, C., Mu, X.J., Khurana, E., Rozowsky, J., Alexander, R.*, et al.* (2012). Architecture of the human regulatory network derived from ENCODE data. Nature *489*, 91-100.

Hezroni, H., Koppstein, D., Schwartz, M.G., Avrutin, A., Bartel, D.P., and Ulitsky, I. (2015). Principles of long noncoding RNA evolution derived from direct comparison of transcriptomes in 17 species. Cell Rep *11*, 1110-1122.

Langmead, B., and Salzberg, S.L. (2012). Fast gapped-read alignment with Bowtie 2. Nat Methods *9*, 357-359.

Moon, M.H., Hilimire, T.A., Sanders, A.M., and Schneekloth, J.S., Jr. (2018). Measuring RNA-Ligand Interactions with Microscale Thermophoresis. Biochemistry *57*, 4638-4643.

Nepal, C., Hadzhiev, Y., Previti, C., Haberle, V., Li, N., Takahashi, H., Suzuki, A.M., Sheng, Y., Abdelhamid, R.F., Anand, S.*, et al.* (2013). Dynamic regulation of the transcription initiation landscape at single nucleotide resolution during vertebrate embryogenesis. Genome Res *23*, 1938-1950.

Pereira, B., Chin, S.F., Rueda, O.M., Vollan, H.K., Provenzano, E., Bardwell, H.A., Pugh, M., Jones, L., Russell, R., Sammut, S.J., *et al.* (2016). The somatic mutation profiles of 2,433 breast cancers refines their genomic and transcriptomic landscapes. Nat Commun *7*, 11479.
Quinlan, A.R., and Hall, I.M. (2010). BEDTools: a flexible suite of utilities for comparing genomic features. Bioinformatics *26*, 841-842.
Rada-Iglesias, A., Bajpai, R., Swigut, T., Brugmann, S.A., Flynn, R.A., and Wysocka, J. (2011). A unique chromatin signature uncovers early developmental enhancers in humans. Nature *470*, 279-283.
Roadmap Epigenomics, C., Kundaje, A., Meuleman, W., Ernst, J., Bilenky, M., Yen, A., Heravi-Moussavi, A., Kheradpour, P., Zhang, Z., Wang, J., *et al.* (2015). Integrative analysis of 111 reference human epigenomes. Nature *518*, 317-330.
Speir, M.L., Zweig, A.S., Rosenbloom, K.R., Raney, B.J., Paten, B., Nejad, P., Lee, B.T., Learned, K., Karolchik, D., Hinrichs, A.S., *et al.* (2016). The UCSC Genome Browser database: 2016 update. Nucleic Acids Res *44*, D717-725.
Waterhouse, A.M., Procter, J.B., Martin, D.M., Clamp, M., and Barton, G.J. (2009). Jalview Version 2--a multiple sequence alignment editor and analysis workbench. Bioinformatics *25*, 1189-1191.
Yue, F., Cheng, Y., Breschi, A., Vierstra, J., Wu, W., Ryba, T., Sandstrom, R., Ma, Z., Davis, C., Pope, B.D., *et al.* (2014). A comparative encyclopedia of DNA elements in the mouse genome. Nature *515*, 355-364.
